# Development of a health behavior scale for older adults living alone receiving public assistance

**DOI:** 10.1186/s12889-021-11347-x

**Published:** 2021-07-19

**Authors:** Ayano Isozaki, Etsuko Tadaka

**Affiliations:** 1 Department of Community Health Nursing, Graduate School of Medicine, Yokohama City University, 3-9 Fukuura, Kanazawa-ku, Yokohama, Kanagawa 236-0004 Japan; 2 Department of Community and Public Health Nursing, Graduate School of Health Sciences and Faculty of Medicine, Hokkaido University, N12-W5, Kita-ku, Sapporo, 060-0812 Japan

**Keywords:** Health behavior, Health disparities, Living alone, Older adults, Scale development

## Abstract

**Background:**

To reduce health disparities, prevention of non-communicable diseases (NCD) by performing desirable health behavior in older adults living alone with low socioeconomic status is an essential strategy in public health. Self-perception of personal power and practical skills for daily health are key elements of desirable health behavior. However, methods for measuring these concepts have not been established. This study aimed to develop a health behavior scale for older adults living alone receiving public assistance (HBSO).

**Methods:**

The self-administered mail survey covered 2818 older adults living alone receiving public assistance (OAP) randomly selected from the list of people receiving public assistance (Seikatsu-hogo in Japanese) at all 1250 local social welfare offices across Japan. Construct validity was confirmed using confirmatory factor analysis. Internal consistency was calculated using Cronbach’s alpha. The self-efficacy for health promotion scale and Health check-up status were administered to assess the criteria-related validity of the HBSO.

**Results:**

In total, 1280 participants (response rate: 45.4%) responded, of which 1069 (37.9%) provided valid responses. Confirmatory factor analysis identified 10 items from two factors (self-perception of personal power and practical skills for daily health) with a goodness of fit index of 0.973, adjusted goodness of fit index of 0.953, comparative fit index of 0.954, and root mean square error of approximation of 0.049. Cronbach’s alpha was 0.75. The total HBSO score was significantly positively correlated with the self-efficacy for health promotion scale (r = 0.672, *p* < 0.001) and the group with health check-up had significantly higher HBSO scores than the group without it (*p* < 0.001).

**Conclusions:**

The HBSO is an easy-to-self-administer instrument that is reliable and valid for OAP. The HBSO could facilitate appropriate assessment of OAP who need to improve their health behavior to prevent NCD, and could be used to determine effective support.

**Supplementary Information:**

The online version contains supplementary material available at 10.1186/s12889-021-11347-x.

## Background

Increasing health disparities is a serious challenge that is growing in developed countries. Lower socioeconomic groups are generally in poorer health and have higher rates of disability and mortality [1, 2]. To reduce health disparities, the importance of targeting older adults from lower socioeconomic groups has been highlighted against the backdrop of a global aging population [3]. Older adults living alone with low socioeconomic status are a particularly important group [4]. In the United States, the number of older adults below the poverty level was nearly 3.7 million in 2008, and has increased to 5.14 million in 2018, with continued growth predicted [5, 6]. Widening economic disparity is a serious challenge in older adults living alone because it contributes not only to increased health disparity but also health inequalities, such as a difference in healthy life expectancy. In Japan, the difference in healthy life expectancy by prefecture is reported to be up to 2.7 years [7], and differences in socioeconomic status have been reported to underlie this situation [8]. Households receiving public assistance tend to be those facing the most difficult socioeconomic conditions. Japan’s public assistance system is aimed at guaranteeing of the minimum standard of living by providing assistance for those who have trouble making a living despite utilizing all their assets and ability [9]. In addition, the system consists of eight types of assistance programs (livelihood, housing, education, medical, long-term care, maternity, occupational, funeral assistance) [9]. According to the Ministry of Health, Labour and Welfare [10] approximately 1.64 million households receive public assistance in Japan (Seikatsu-hogo in Japanese), 55.3% of the total number of households are those of older adults, accounting for the largest proportion of the total. Furthermore, more than 90% of these older adults’ households (approximately 830,000 households) are those in which individuals live alone. In addition, more than 90% of older adults’ households receiving public assistance receive medical assistance [11]. Thus, older adults who live alone comprise the majority of public assistance recipients, representing a vulnerable population that commonly require medical assistance and face high rates of both daily living risks and chronic illness risks. Thus, older adults living alone receiving public assistance are the most important target group for addressing health disparity in Japan. This group is hereinafter referred to as OAP.

Non-communicable diseases (NCD) account for a large proportion of chronic illnesses, and constitute one of the most important health challenges for reducing health disparities. The World Health Organization (WHO) [12] explains NCD as cardiovascular diseases, cancers, chronic respiratory diseases and diabetes caused by inappropriate health behavior. The WHO [12] also reported that NCD kill 41 million people each year, accounting for 71% of all deaths worldwide. It has been pointed out that socioeconomic factors are closely related to this background, and the more socio-economically disadvantaged a person is, the more likely they are to develop NCD and become severely ill [12]. Among OAP in Japan, NCD represent the main target of medical assistance, with cardiovascular diseases accounting for the largest share (29%) of the total [13]. Thus, prevention of NCD is critical for reducing health disparities in OAP.

Health behavior is a key concept in the prevention of NCD. NCD are considered to be preventable with appropriate health behavior [14]. As one of the definitions of health behavior, Gochman [15] defined health behavior as not only actions, behavioral patterns, and habits which appear on the surface, but also personal attributes related to health maintenance and wellness, restoration, and health improvement. Previous studies reported that desirable health behavior among older adults can lower the risk of heart failure [16], prevent the development of diabetes [17], and lower the risk of dying from cancer [18]. Thus, it is clear that health behavior has a positive impact on NCD prevention during the aging process, and improving appropriate health behaviors is important for NCD prevention, even among older adults.

However, older adults with low socioeconomic status, including OAP, have been reported to have difficulty performing health behaviors that are generally considered desirable [19, 20]. A previous study reported that low socioeconomic status groups are less likely to engage in health behaviors considered desirable because of two types of factors: internal factors and external factors [21]. A internal characteristic in OAP is a lack of personal power that manifests as a reduction in positive feelings (e.g., low self-esteem, self-usefulness, and self-affirmation); this characteristic leads to poor health and is shaped by socioeconomic background [22–25]. Being in a socio-economically difficult situation exposes individuals to everyday stresses, as well as discrimination and prejudice [26]. These experiences rob OAP of perception of their power because they create negative feelings [26, 27]. This discourages individuals from looking at themselves and making healthy choices, which in turn makes it difficult to perform appropriate health behaviors [26, 28–30]. Therefore, it is important for OAP to be able to recognize the self-perception of personal power. The characteristics of external factors in OAP include a lack of skills for good practice for health behavior consisting of knowledge and experience [31, 32]. The scarcity of knowledge and experience that constitute skills for good practice results from a lack of opportunities to acquire them, and the inaccessibility of those opportunities [32, 33]. This prevents individuals from undertaking actions that are good for their health, and makes it difficult for them to carry out those actions continuously and effectively [20, 34]. Therefore, it is important for OAP to develop practical skills for daily health. Link and Phelan [35] stated that health disparities are caused by differences in the resources available to people according to their socioeconomic status. They also point out that people with low socioeconomic status have less access to health promotion resources and, as a result, lack the opportunity to develop the personal power and practical skills that are key components of healthy behavior. On the basis of the factors described above, “self-perception of personal power” and “practical skills for daily health” can be considered essential components of specific health behavior in OAP.

Because health behavior for NCD prevention has long been an important issue internationally, many measures of health behavior have been developed [36–45]. However, all of these scales target general adults and older adults, and their measurement contents are mainly focused on the health behaviors themselves, such as diet and physical activity. Thus, existing scales are limited because they do not target OAP, and it is difficult to capture contents other than health behaviors themselves, such as diet and exercise. This makes it difficult to elucidate important underlying factors, such as self-perception of personal power and practical skills for daily health, which are specific to OAP health behavior. To the best of our knowledge, no scales for NCD prevention among OAP have been developed. Thus, a new scale is needed based on a concept of health behavior that encompasses self-perception of personal power and practical skills for daily health. This new scale would allow appropriate assessment of OAP health behaviors and effective support based on such assessment.

In the current study, we developed the “Health behavior scale for older adults living alone receiving public assistance” (HBSO), an instrument for assessing health behavior for the prevention of NCD in OAP, and tested its reliability and validity.

## Methods

### Phase 1: developing the instrument

First, we developed a pool of items based on a literature review. From the perspective of OAP health behavior, we searched PubMed, Web of Science, CiNii, and Ichushi-web for related articles. We used specific keywords: health behavior; health practice(s); healthy lifestyle; older adults; poverty; socio-economic status; disadvantaged people; and living alone. This search identified 17 articles [4, 33, 34, 36–49]. On the basis of this review, we defined health behavior as personal attributes, characteristics, and actions that lead to NCD prevention in OAP. With reference to previous studies, a pool of items was based on four perspectives: 1) Items that are considered to be particularly necessary or important for the maintenance and promotion of the health among OAP; 2) Items that are not impossible to implement for economic reasons; 3) Items that reflect characteristics that place older adults living alone at higher risk than older adults living with family members (e.g., social isolation, lack of emergency assistance, unbalanced diet, tobacco smoking); and 4) Items that are practically beneficial. Considering these four points, we reviewed the pool of draft items and made several modifications; the result was a final list of 34 items.

Second, the item pool was reviewed by three OAP, three professionals, and two researchers to assess the content validity and face validity of the items. The three OAP were 65–70 years old. The professionals comprised a public health nurse and two visiting nurses from a health center. The researchers comprised a professor and one assistant professor from the Department of Community Health Nursing who specialized in community health nursing. In this process, the modified HBSO scale was refined to 20 items.

### Phase 2: validating the instrument

#### Participants and settings

The self-administered mail survey covered 2818 OAP who were randomly selected from the list of public assistance (Seikatsu-hogo in Japanese) at all 1250 local social welfare offices across Japan. Informed consent for this study was obtained in two steps. The aim of these two steps was to secure the required sample size despite the fact that the researchers could not directly access the OAP for reasons of privacy protection. First, we sent a letter to the offices explaining the purpose of the study, and requesting cooperation with the study. As a result, consent for the study was obtained from 155 offices (12.5%). Next, staff from 155 offices randomly distributed questionnaires to a total of 2818 OAP. The questionnaire asked participants to reflect on their behavior and answer the questions themselves. However, we informed participants that if they found it difficult to fill in the questionnaire, they could communicate their responses to social welfare office staff or other individuals, who would fill in the questionnaire for them. As a result, 1280 (45.4%) participants responded to the questionnaire, of which 1069 (37.9%) provided valid responses suitable for analysis. The criteria for valid responses were aged ≥65, no or one missing HBSO item response, and no missing responses for the self-efficacy for health promotion scale and health check-up status, which were used as validity indicators. To check for bias when participants who did not provide valid responses were excluded, we compared data from participants with valid responses versus those without valid responses. A total of 211 respondents did not provide valid responses; the mean age was 73.2 ± 8.0 years and 54.0% were female. The results were similar to those for respondents with valid responses (mean age of 74.5 ± 6.7 years, 52.9% female), so we concluded that the exclusion did not bias the data. Data were collected between August and September 2020.

#### Measures

Participants’ demographic data included: age, sex, certification for long-term care needs (Support need level 1 and 2, and Care need level 1 to 5; a larger number indicates a more severe level) under the Long-Term Care Insurance system in Japan, period of public assistance, illness under treatment, Status of health check-up in the last year, alcohol drinking habits, and smoking history (Table 1).

Participants were asked to complete the modified 20-item version of the HBSO. Each item was assessed on a 4-point Likert-type scale: 0 = disagree, 1 = disagree to a certain extent, 2 = agree to a certain extent, and 3 = agree. Missing data were treated as follows. One missing value was substituted with the average value for the other items [50, 51]. If more than one item was missing, the response to that questionnaire was considered invalid. We used this missing complement method because we were concerned that the participant characteristics would cause strong selection bias in a complete-case analysis.

To assess criteria-related validity of the HBSO, we used one measure and one indicator. First, we used the self-efficacy for health promotion scale [52]. The self-efficacy for health promotion scale comprises 15 items belonging to one factor. Items are scored from 1 to 4 (range 15–60), with higher scores indicating higher self-efficacy in health care among older adults, predictive of the implementation of appropriate health promotion behaviors. The self-efficacy for health promotion scale had a Cronbach’s alpha of 0.89. Second, we used health check-up status. Since a high total score on the HBSO indicates that respondents took actions to benefit their health, health check-up status was selected as an objective health indicator. Responses to the question “Have you had a regular health check-up in the last year?” were scored as follows: “Did receive = 1 point”; “Did not receive = 0 points”.

#### Statistical analysis

We conducted all analyses using IBM SPSS Statistics 25.0 and Amos 25.0. Item analyses were conducted to ensure that only pertinent, functional, and internally consistent items were included. The criteria for item analysis included rates of difficulty (non-respondents ≥5%), distribution (after “agree” and “agree to a certain extent” were over 90%), correlations between each item (correlation coefficient > 0.45), item-total analysis (correlation coefficient < 0.3), and good-poor analysis (no significant differences between the highest-and lowest-scoring groups).

After item analysis, we randomly divided the total sample (*n* = 1069) into two sub-samples for cross-validation: group 1 (*n* = 535) was used for performing exploratory factor analysis; and group 2 (*n* = 534) was used for performing confirmatory factor analysis. The items remaining after item analysis were examined using exploratory factor analysis (maximum likelihood method) with promax rotation. The optimal number of factors was determined by sequentially using latent root criteria (eigenvalues > 1.0) and a scree plot. Item loadings needed to exceed 0.40. Confirmatory factor analysis was then conducted to verify the construct validity. The goodness-of-fit index (GFI), adjusted goodness-of-fit index (AGFI), comparative fit index (CFI), and root-mean-square error of approximation (RMSEA) were used to evaluate the data model fit. The model was accepted if the GFI, AGFI and CFI were ≥ 0.90, and RMSEA was ≤0.05 [53, 54]. Cronbach’s alpha was used to evaluate the internal consistency of the HBSO, with a value of ≥0.70 considered adequate. Furthermore, criteria-related validity was examined using the self-efficacy for health promotion scale and the health check-up status indicator.

## Results

### Demographic characteristics

In total, 1280 participants (response rate: 45.4%) responded to the questionnaire, of which 1069 (37.9%) provided valid responses suitable for analysis. Table 1 shows the demographic characteristics of OAP. The mean age was 74.5 years. Overall, 52.9% of participants were female, and 66.3% were not certified for long-term care insurance. The mean period of public assistance was 8 years and 6 months, and the mean number of illnesses under treatment was 1.4. of participants, 50.7% had a health check-up in the last year, 23.3% had a drinking habit, and 56.4% had a smoking history (Table 1). Furthermore, there were geographical differences in the locations of the welfare offices that agreed to participate; the Chubu region, which is one of the seven regional divisions of Japan, had the largest proportion of participants (33.7%).

**Table 1:** Participants’ demographic characteristics.

		Total (*n* = 1069)		Group 1 ^a^ (*n* = 535)		Group 2 ^b^ (*n* = 534)	
		Number or Mean ± SD ^c^	% or (Range)	Number or Mean ± SD ^c^	% or (Range)	Number or Mean ± SD ^c^	% or (Range)
Age, years		74.5 ± 6.7	(65–98)	74.6 ± 6.7	(65–98)	74.4 ± 6.6	(65–96)
	65–69	280	26.2	141	26.4	139	26.0
	70–74	294	27.5	149	27.9	145	27.2
	75–79	236	22.1	113	21.1	123	23.0
	80 ≥	227	21.2	120	22.4	107	20.0
	Missing	32	3.0	12	2.2	20	3.7
Sex	Female	565	52.9	274	51.2	291	54.5
	Missing	13	1.2	8	1.5	5	0.9
Certification for long-term care insurance	No/independence	709	66.3	358	66.9	351	65.7
	Support need levels 1 and 2	128	12.0	59	11.0	69	12.9
	Care need levels 1 and 2	64	5.9	30	5.6	34	6.4
	Care need levels 3–5	23	2.2	12	2.2	11	2.1
	Missing	145	13.6	76	14.2	69	12.9
Period of receiving public assistance		8.6 ± 6.6 ^d^	(1 M-61Y)^e^	8.6 ± 6.7 ^d^	(1 M-41Y) ^e^	8.7 ± 6.6 ^d^	(2 M-61Y) ^e^
	< 1 year	37	3.5	20	3.7	17	3.2
	1 years ≥,and 5 years<	246	23.0	122	22.8	124	23.2
	5 years ≥,and 10 years<	323	30.2	167	31.2	156	29.2
	10 years ≥,and 15 years<	219	20.5	99	18.5	120	22.5
	15 years ≥	155	14.5	80	15.0	75	14.0
	Missing	89	8.3	47	8.8	42	7.9
Illness under treatment		1.4 ± 1.0	(0–7.0)	1.4 ± 1.0	(0–5.0)	1.4 ± 1.0	(0–7.0)
	Yes	876	81.9	433	80.9	443	83.0
	Hypertension	445	41.6	216	40.4	229	42.9
	Diabetes	218	20.4	99	18.5	119	22.3
	Heart disease	154	14.4	82	15.3	72	13.5
	Respiratory disease	104	9.7	55	10.3	49	9.2
	Dyslipidemia	77	7.2	27	5.0	50	9.4
	Cancer	65	6.1	38	7.1	27	5.1
	Others	378	35.3	176	32.9	202	37.8
	Missing	37	3.5	22	4.1	15	2.8
Health check-up in the last one year	Yes	542	50.7	267	49.9	275	51.5
	No	527	49.3	268	50.1	259	48.5
Alcohol drinking habits	Yes	249	23.3	115	21.7	134	25.1
	Missing	46	4.3	21	3.9	25	4.7
Smoking history	Yes	603	56.4	304	56.8	299	56.0
	Missing	26	2.4	15	2.8	11	2.1

^a^ Group 1 was used for performing exploratory factor analysis

^b^ Group 2 was used for performing confirmatory factor analysis

^c^ SD, standard deviation. ^d^ Unit: Year. Month. ^e^ M: Month, Y: Year

### Item analysis

Table 2 shows the item analysis results. Six items (items 4, 5, 13, 14, 15, and 18) met the exclusion criteria for inter-item correlation. However, we retained items 4, 13, and 14. One item (item 6) met the exclusion criteria for item total correlation. Item 10 was then excluded because “Health check-up status” was adopted as an indicator of criteria-related validity.

**Table 2:** Item analysis of the HBSO

No. Item	Item distribution^b^	Population difficulty^a^	Inter-Item Correlation^c^	Good-Poor Analysis^d^	Item-Total Correlation^e^		Exclusion
(*N* = 1069)							
1 I make sure to brush my teeth after every meal.	0.1	65.1	–	***	.346	**	
2 I wash my hands and gargle regularly to protect against infection.	0.0	87.8	–	***	.381	**	
3 I go to bed and get up at around the same time every day.	0.2	83.8	–	***	.319	**	
4 I do not drink alcohol. Or if I do, I make sure not to drink excessively.	0.7	80.4	+	***	.324	**	
5 I make sure not to smoke.	0.3	67.0	+	***	.346	**	×
6 I make sure to not sweet bread and snacks as much as possible.	0.3	58.7	–	***	.281	**	×
7 I choose foods by checking information such as nutritional value, salt and calories.	0.3	55.0	–	***	.398	**	
8 I move my body as much as possible every day to maintain an appropriate level of exercise.	0.1	65.6	–	***	.411	**	
9 I gather information that helps me stay healthy from articles, TV programs and others.	0.3	55.4	–	***	.407	**	
10 I have regular health check-ups with or without any signs or symptoms.	0.5	54.7	–	***	.324	**	×
11 When I have dental problems, I do not leave them and go to see the dentist as soon as possible.	0.7	54.1	–	***	.392	**	
12 Even when feeling frustrated, I make sure not to vent off by binge-drinking, smoking or binge-eating.	0.2	74.0	–	***	.406	**	
13 I have my own ways to distract or change my mind.	0.2	69.7	+	***	.490	**	
14 I have someone that I can talk to comfortably if I need to.	0.2	64.5	+	***	.482	**	
15 I make sure to ask for help when I face issues that I cannot resolve on my own.	0.4	60.5	+	***	.470	**	×
16 I make sure to consult with health and welfare professionals when I have problems in my health and life.	0.2	54.8	–	***	.395	**	
17 I have places where I can relax besides home.	0.3	37.0	–	***	.405	**	
18 I have hobbies and activities that help me enjoy daily life.	0.3	55.9	+	***	.429	**	×
19 I spend my time trying to help others even in a small way.	0.1	50.4	–	***	.491	**	
20 I have goals and hopes for the future of my life.	0.0	38.2	–	***	.472	**	

***: *p* < 0.001, **: *p* < 0.01

Exclusion criteria for the item analysis

^a^: The percentage of non-respondents is over 5% of the sample

^b^: Percentage of ‘agree’ and ‘agree to a certain extent ‘is over 90% of the sample

^c^: Correlation between each item is over 0.45

^d^: Difference of the average score between the highest-and lowest-scoring groups is not significant difference (*p* ≥ 0.05)

^e^: Correlation coefficient between the item and the total of all the items (but with exception of the items) is less than 0.3

Notes

Item 10 was excluded. Because “The status of health check-ups” was adopted as an indicator of criterion-related validity

Thus, five items (items 5, 6, 10, 15, and 18) were excluded and 15 items (items 1–4, 7–9, 11–14, 16–17, and 19–20) were subjected to factor analysis.

### Factor structure

The results of exploratory factor analysis are shown in Table 3. The eigenvalues were 3.758 for one factor, 1.393 for two factors, and 1.323 for three factors. Eigenvalues and scree plots suggested a one-factor or two-factor model. We repeated exploratory factor analysis with promax rotation until the factor loadings exceeded 0.4. The difference in factor loadings between each factor became clear, and the factor became theoretically most explicable. As a result, we excluded items 3, 4, 8, 12 and 16 because the factor loading did not exceed 0.4 in any analysis. Excluding items with a loading of less than 0.4 resulted in a two-factor solution. We extracted 10 items for two factors for the final version of the scale. Factor 1 included five items (items 17, 14, 13, 19, and 20) interpreted as “Self-perception of personal power”, reflecting individuals’ perception of their own power. Factor 2 included five items (items 1, 7, 2, 9, 11), interpreted as “Practical skills for daily health”, reflecting skills related to practicing healthy living one day at a time. The factor loadings were greater than 0.4 for each factor. The cumulative contribution of the two factors explained 30.1% of the variance. Furthermore, the correlation coefficient between the two factors was 0.53.

**Table 3:** Exploratory factor analysis of the HBSO

*n* = 535				
No.	Item	Factor I	Factor II	Total scale communality
		Self-perception of personal power	Practical skills for daily health	
17	I have places where I can relax besides home.	**0.70**	−0.19	0.28
14	I have someone that I can talk to comfortably if I need to.	**0.62**	−0.01	0.30
13	I have my own ways to distract or change my mind.	**0.49**	0.10	0.28
19	I spend my time trying to help others even in a small way.	**0.47**	0.11	0.27
20	I have goals and hopes for the future of my life.	**0.43**	0.21	0.21
1	I make sure to brush my teeth after every meal.	−0.08	**0.57**	0.31
7	I choose foods by checking information such as nutritional value, salt and calories.	−0.03	**0.54**	0.37
2	I wash my hands and gargle regularly to protect against infection.	0.04	**0.53**	0.39
9	I gather information that helps me stay healthy from articles, TV programs and others.	0.01	**0.52**	0.28
11	When I have dental problems, I do not leave them and go to see the dentist as soon as possible.	0.07	**0.42**	0.32
Cumulative contribution (%)		23.3	30.1	
Factor correlation coefficients (r)	Factor I	1.00	0.53	
	Factor II	0.53		

Maximum likelihood method factor analysis with promax rotation

Bold: Item loadings exceed 0.40

### Internal consistency and validity

**Fig. 1:**
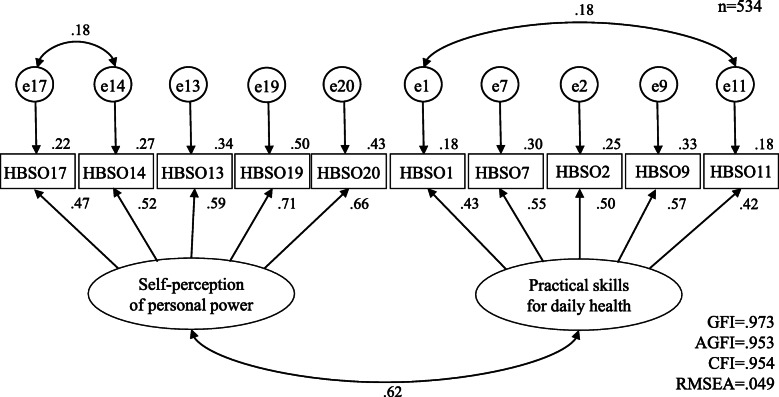
Confirmatory factor analysis of the HBSO(final version)

Cronbach’s alpha coefficients were 0.72 for factor 1, 0.63 for factor 2, and 0.75 for the total scale (Table 4). These two factors were entered as latent factors in a confirmatory factor analysis model. In the initial model, GFI = 0.964, AGFI = 0.942, CFI = 0.927, and RMSEA = 0.060, which did not represent a good data-model fit. The model fit improved after modifying the model according to modification indices, adding error correlations for items 17 and 14, and items 1 and 11, as well as incorporating the improved factors: GFI = 0.973, AGFI = 0.953, CFI = 0.954, and RMSEA = 0.049. These results satisfied the appropriate criteria in all subjects (Fig. 1).

**Table 4:** Criteria-related validity of the HBSO

*n* = 1069						
Factors	Mean (SD ^a^)	Self efficacy for health promotion scale	Health check-up status			Chronbach’s alpha
			Yes	No	Mean difference	
I: Self-perception of personal power	7.52 (3.83)	0.622***	8.14(3.83)	6.89(3.73)	−1.25***	0.72
II: Practical skills for daily health	9.02 (3.42)	0.493***	9.65(3.26)	8.37(3.46)	−1.28***	0.63
Total 10 items	16.54 (6.06)	0.672***	17.79(5.82)	15.26(6.04)	−2.53***	0.75

^a^SD: standard deviation

***: *p* < 0.001

Pearson’s correlation analysis revealed correlations between total HBSO scores and total scores of the self-efficacy for health promotion scale. We also examined differences in HBSO scores by the presence of health check-up status, revealing a significant difference. HBSO scores exhibited significantly positive correlation with self-efficacy for health promotion scale scores (r = 0.672, *p* < 0.001). Mean HBSO scores were 17.8 (SD: 5.8) in the group with health check-up and 15.3 (SD: 6.0) in the group without health check-up. The group with health check-up had significantly higher HBSO scores than the group without health check-up (p < 0.001) (Table 4). Furthermore, we also checked HBSO scores for drinking habits and smoking history, which are strongly associated with NCD. The mean HBSO scores were 17.0 (SD: 6.0) in the group with no drinking alcohol habit and 15.1 (SD: 6.1) in the group with drinking alcohol habit. Participants in the no drinking alcohol habit group had significantly higher HBSO scores than those in the drinking alcohol habit group (*p* < 0.001). Mean HBSO scores were 18.2 (SD: 5.7) in the group with no smoking history and 15.3 (SD: 6.0) in the group with smoking history. The group with no smoking history had significantly higher HBSO scores than the group with smoking history (p < 0.001).

## Discussion

The participants for this study were drawn from a national sample of OAP in Japan. A comparison between the participants in this study and demographic data from National survey on public assistance [11] is as follows. Our participants were similar in age composition, with a mean age of 74.5 years in the current study, compared with 75.5 years in the previous survey. The percentage of females was 52.9% in the current study, compared with 52.1% in the previous survey. Thus, our study sample appeared to be relatively representative of the wider population of OAP in Japan.

The HBSO has two main original features. First, we developed a new instrument for using with OAP to support improving health behavior preventing NCD. The HBSO is a two-factor, 10-item instrument that is easy to self-administer. The results revealed that the HBSO was sufficiently reliable and valid. Confirmatory factor analysis of the model was used to validate the factors (GFI = 0.973, AGFI = 0.953, CFI = 0.954, RMSEA = 0.049). Regarding the reliability of the HBSO, Cronbach’s alpha indicated sufficient internal consistency. Criterion-related validity showed that HBSO and self-efficacy for health promotion scale were significantly positively correlated (r = 0.672, *p* < 0.001), and t-test results revealed that HBSO scores were significantly higher in the group that received a health check-up compared with the group that did not (p < 0.001). Furthermore, we found that the group without drinking habits or smoking history had significantly higher HBSO scores than the group with drinking habits and smoking history (p < 0.001). Therefore, the HBSO developed in this study was determined to be a sufficient, reliable, and valid measure enabling the evaluating and supporting of health behaviors in OAP to prevent NCD. Second, we developed a concept of health behavior in OAP, consisting of “self-perception of personal power” and “practical skills for daily health”. A key difference between concept of HBSO and previous health behavior measures is that previous scales [36–45] have mainly focused on overt actions of general adults in a stable socioeconomic state toward health promotion and care prevention. In contrast, the HBSO focuses on OAP, revealing that OAP health behavior to prevent NCD is composed of elements that may not be easily observable, such as perception and skills. Williams et al. [39] pointed out the need for health behavior scales that consider socioeconomic background. HBSO will enable the assessment of previously latent elements in relation to socioeconomic status, such as self-perception of personal power and practical skills for daily health. This scale used self-report items that were easy for the OAP to understand and to answer; therefore, it could help OAP who have few opportunities to engage in healthy behaviors to self-reflect on their lives and their health. In particular, the scale items could prompt OAP to consider ways to improve their health, and to identify specific aspects for improvement by comparing the health behaviors described in the items to their daily lives. The scale items not only highlight what is lacking, but also draw attention to existing strengths; if used regularly, the scale could help OAP to identify positive changes in health behavior. We believe that the HBSO can capture small changes made by OAP under difficult conditions. The accumulation of all these small changes would create a bigger change. We believe that the HBSO is a first step in self-motivating OAP to improve their health.

The first factor of the HBSO, “Self-perception of personal power” includes items that reflect positive feelings engendered by social relationships, which help to generate good health. Prior research has reported that recognizing personal power is a key component of health behavior [28, 30, 55]. However, OAP have been reported to lack perceived personal power [22–25]. Therefore, self-perception of personal power is important as a health behavior in OAP because it enables OAP to look at themselves and make better choices for their health [26, 28–30].

The second factor in the HBSO, “Practical skills for daily health” includes items that reflect personal ability for practicing healthy living one day at a time. Basic cleanliness, nutrition, and disease prevention skills are foundational to practicing other, more extensive health behaviors, but prior research suggests that OAP are less likely to acquire these skills [31, 32]. Therefore, practical skills for daily health are important as OAP health behaviors because it enable OAP to practice them more effectively and continuously in their daily lives [20, 33, 34].

Because the current study involved the limitation of a cross-sectional design, the predictive validity of the results is unclear. In future, it will be necessary to conduct a longitudinal study to determine whether higher HBSO scores are associated with the prevention of NCD.

## Conclusion

The HBSO is a reliable and valid instrument. This scale uses a self-report format that is easy for OAP to understand and respond to. Therefore, it can help OAP with few opportunities to engage in health behaviors to self-reflect on their lives and their health. We demonstrated in this study that the concept of health behavior for OAP consists of the self-perception of personal power and practical skills for daily health. The HBSO could help to identify small changes created by individual efforts under difficult conditions; such changes have not been previously measured. The HBSO could facilitate the appropriate assessment of OAP who need to improve their health behavior to prevent NCD, and could be used to determine effective support for OAP. Enhancement of the two aspects of OAP health behaviors identified in this study is the first step toward helping OAP to engage in self-care to prevent NCD. This in turn will contribute to reducing health disparities.

## Supplementary Information

**Additional file 1.** English version of the final HBSO.

**Additional file 2.** Japanese version of the final HBSO.

## Data Availability

The datasets generated and analyzed during the current study are not publicly available because the Ethical Guidelines for Epidemiological Research by the Japanese Government and the National Basic Resident Registration System administered by the Ministry of Internal Affairs and Communications in Japan prohibit researchers from providing their research data to other third-party individuals but are available from the corresponding author on reasonable request.

## Abbreviations

AGFI: Adjusted goodness-of-fit index
CFI: Comparative fit index
GFI: Goodness-of-fit index
HBSO: Health behavior scale for older adults living alone receiving public assistance
NCD: Non-communicable diseases
OAP: Older adults living alone receiving public assistance
RMSEA: Root-mean-square error of approximation
