# Supplementary material for: Development of a health behavior scale for older adults living alone receiving public assistance
Source: BMC Public Health. 2021 Jul 19;21:1428. doi: 10.1186/s12889-021-11347-x (PMC8290590; doi:10.1186/s12889-021-11347-x)
Supplement: Supplementary file 1 — Additional file 1. English version of the final HBSO. [file 12889_2021_11347_MOESM1_ESM.pdf]

Additional file 1: English version of the final HBSO

Health behavior scale for older adults living alone receiving public assistance (HBSO)

Evaluation

0: Disagree 1: Disagree to a certain extent 2: Agree to a certain extent 3: Agree

No.	Domain & Item	Evaluation			
**Self-perception of personal power**					
1	I have places where I can relax besides home.	0	1	2	3
2	I have someone that I can talk to comfortably if I need to.	0	1	2	3
3	I have my own ways to distract or change my mind.	0	1	2	3
4	I spend my time trying to help others even in a small way.	0	1	2	3
5	I have goals and hopes for the future of my life.	0	1	2	3
**Practical skills for daily health**					
6	I make sure to brush my teeth after every meal.	0	1	2	3
7	I choose foods by checking information such as nutritional value, salt and calories.	0	1	2	3
8	I wash my hands and gargle regularly to protect against infection.	0	1	2	3
9	I gather information that helps me stay healthy from articles, TV programs and others.	0	1	2	3
10	When I have dental problems, I do not leave them and go to see the dentist as soon as possible.	0	1	2	3
	**No.1-10 total**	**point**			

Isozaki (Inoue) A, Tadaka E: Development of a health behavior scale for older adults living alone receiving public assistance, BMC Public Health.
