# Supplementary material for: Development of a health behavior scale for older adults living alone receiving public assistance
Source: BMC Public Health. 2021 Jul 19;21:1428. doi: 10.1186/s12889-021-11347-x (PMC8290590; doi:10.1186/s12889-021-11347-x)
Supplement: Supplementary file 2 — Additional file 2. Japanese version of the final HBSO. [file 12889_2021_11347_MOESM2_ESM.pdf]

Additional file 2: Japanese version of the final HBSO

Health behavior scale for older adults living alone receiving public assistance (HBSO)

生活保護を受給している独居高齢者の保健行動尺度(HBSO)

以下の項目について、あなたのふだんの生活に、最も近い数字１つに○をつけてください。

領域／項目		あてはまらない	やや<br>あてはまらない	やや<br>あてはまる	あて<br>はまる
**自分自身のパワーの認識**					
1	私には、家以外にもほっとできる身近な居場所がある	0	1	2	3
2	私には、ふだんから気軽に相談できる相手がいる	0	1	2	3
3	私には、気晴らしや気分転換ができる自分なりの方法がある	0	1	2	3
4	私は、小さなことでも人の役に立てるよう過ごしている	0	1	2	3
5	私には、この先の生活の目標や希望がある	0	1	2	3
**日々の健康に向けた実践的スキル**					
6	私は、食事の後には毎回、歯をみがくようにしている	0	1	2	3
7	私は、栄養成分、塩分、カロリーなどを確認して、食べ物を選んでいる	0	1	2	3
8	私は、こまめに手洗いうがいをして、感染症を予防している	0	1	2	3
9	私は、記事やテレビ番組などで、健康に良い情報を集めている	0	1	2	3
10	私は、歯の調子が悪い時には、放っておかず、早めに受診している	0	1	2	3
**No.1-10 合計**		**点**			

Isozaki (Inoue) A, Tadaka E: Development of a health behavior scale for older adults living alone receiving public assistance, BMC Public Health.
